# Association Between Pet Keeping and Current Asthma Among Adolescents Living in Yazd; Evidence from Global Asthma Network (GAN) 2020 Cross-sectional Study

**DOI:** 10.34172/aim.2023.102

**Published:** 2023-12-01

**Authors:** Nasrin Behniafard, Seyedeh Zalfa Modarresi, Zahra Nafei, Mahmood Vakili

**Affiliations:** ^1^Children Growth Disorder Research Center, Shahid Sadoughi University of Medical Sciences, Yazd, Iran; ^2^Health Monitoring Research Center, School of Medicine, Shahid Sadoughi University of Medical Sciences, Yazd, Iran

**Keywords:** Adolescent, Allergy, Asthma, Pets

## Abstract

**Background:**

The relationship between current pet keeping and allergic diseases, including bronchial asthma in adolescents, is controversial. This study was conducted to evaluate these associations among children aged 13-14 years in Yazd.

**Methods:**

This study is part of a multicenter cross-sectional study of the Global Asthma Network (GAN) in Yazd, Iran, in 2020, in which 5141adolescents enrolled. Information on respiratory symptoms and pet-keeping (dog/cat/birds) was obtained by a questionnaire derived from the GAN standard questionnaire.

**Results:**

Of 5141 participants who completed the study, 1800 (35%) children kept pets during the last year. Birds were the most common pet kept by adolescents (88%). Severe asthma was more common in bird and cat keepers (*P*=0.003 and *P*=0.034, respectively) than dog keepers. Furthermore, there was a statistically significant association between study-defined current asthma and cat keeping, but not bird or dog ownership (*P*=0.02). Moreover, we found that current any pet-keeping (birds, cats, dogs) was associated with a higher prevalence of asthma-related symptoms, including wheezing, night dry cough, and exercise-induced wheezing in the past year (*P*=0.002, *P*=0.000 and *P*=0.000 respectively)

**Conclusion:**

Current any pet-keeping is associated with asthma-related symptoms. Additionally, cat keeping had a significant association with study-defined current asthma. The current keeping of birds, as the most common pet in our area, or cat keeping increases the risk of severe asthma in adolescents. Therefore, as an important health tip, this needs to be reminded to families by health care providers.

## Introduction

The prevalence of asthma in children and adolescents has increased in recent decades, estimated at 14.1% according to phase III of the International Study of Asthma and Allergies in Childhood (ISAAC) conducted in 97 countries.^1,2^ In Iran, the total prevalence of asthma has been recorded at 10.9% among the teenage population.^3^

Based on the Global Asthma Prevention Initiative (GINA), pet keeping is identified as a significant source of animal allergens, endotoxins, and microbes. This exposure is considered a potential environmental risk factor for the development of childhood allergic disorders, including asthma and wheezing.^4-6^

The socio-economic development has led to more children having the chance to interact with pets at home.^7^ However, the relationship between keeping pets and asthma symptoms is not consistent across studies. Some research suggests that having pets can increase the risk of asthma, while others find that exposure to pets early in life can decrease the risk of asthma. Additionally, some studies have not found any significant link between keeping pets and asthma.^8-12^

It seems that the increased levels of allergens in the air released by saliva, skin, and hair follicles of pets, or the increased levels of endotoxins in indoor air, may have a protective effect on asthma.^13,14^ In addition, the age at which children come in contact with pets has conflicting results in terms of the risk of developing asthma. As shown, the risk of developing asthma and wheezing in children over six years exposed to pets is higher than in children under six years of age. Furthermore, a protective effect has been detected in some research, in which exposure is assessed shortly before outcome.^15^

Another issue to be known is that the role of exposure to pets in childhood asthma varies with the geographic region because children tend to become sensitized to local allergen.^16^

 As part of the Global Asthma Network (GAN) survey, this cross-sectional study aimed to explore the prevalence of pet-keeping in adolescents in Yazd, Iran, and evaluate the associations between pet keeping and asthma and asthma-related symptoms.

## Materials and Methods

###  Subjects

This cross-sectional study was performed as part of the GAN study to investigate the relationship between pet keeping and study-defined current asthma and asthma-related symptoms in adolescents during 2020 in Yazd. Forty-eight public and private schools in urban areas of Yazd and from both educational districts were selected by simple random sampling. Schools with non-Iranian students were excluded from the study. All students aged 13-14 years from selected schools were asked to complete the questionnaire. Due to the COVID-19 pandemic and closure of schools, school children were asked to fill an electronic questionnaire based on the GAN standard questionnaire that was translated to Farsi and validated. More details about this study have been published elsewhere.^17^

 Out of 7241 school children, 5141 completed the questionnaire (71.3%). After written consent was obtained, the children were asked to complete the questionnaire, as participation was strictly voluntary. In the present study, data from all 5141 GAN study participants were analyzed.

###  Questionnaire Data

 We used the standardized written questionnaire of GAN to assess the children’s respiratory health and exposure to pets including dogs and cats. We also added a question on bird keeping in our study.

 The questions about pet keeping addressed current (past 12 months) pet-keeping by asking the following questions:

1. In the past 12 months, have you had a cat in your home?
2. In the past 12 months, have you had a dog in your home?
3. In the past 12 months, have you had a bird in your home? (This question was added to the original GAN questionnaire)

###  Asthma Symptoms 

 According to the previous GAN methodology, the prevalence of current asthma symptoms was estimated based on the affirmative responses to the following questions:

a) Have you had wheezing or whistling in the chest in the past 12 months? (current wheezing)
b) In the past 12 months, has your chest sounded wheezy during or after exercise?
c) In the past 12 months, have you had a dry cough at night, apart from a cough associated with a cold or chest infection?

Current asthma (asthma in the past 12 months) was identified by a positive response to the following questions:

d) “Was asthma confirmed by a doctor?”

And each of the following:

e) “Have you had wheezing or whistling in the chest in the past 12 months?”

or

f) “Have you used any inhaled medicines, e.g. puffers (using local terminology) to help your breathing problems at any time in the past 12 months (when you did not have a cold)?”

Furthermore, “severe asthma” was defined as a combination of wheezing attacks, occurring more than 4 times in the past 12 months, or sleep disturbance for more than 1 night per week or speech-limiting wheezing with asking the following questions:

g) How many attacks of wheezing have you had in the past 12 months?
h) In the past 12 months, how often, on average, has your sleep been disturbed due to wheezing?
i) In the past 12 months, has wheezing ever been severe enough to limit your speech to only one or two words at a time between breaths?

The other questions asked in the questionnaire included in the questions about medications used, including:

 Have you used any inhaled medications, e.g. puffers to help breathing problems at any time in the past 12 months (when you did not have a cold)?

###  Statistical Analysis

Data analyses were done using SPSS version 23.0 (IBM, Armonk, NY). Pearson chi-square test was used to determine the relationship between variables that were expressed as percentages. Statistically, a significant *P* value was considered as < 0.05. As sampling was done by school, while the information was gained from the school pupils, there is likely to be a cluster effect. The sample size of this study was sufficiently large to allow good power in the presence of moderate intra-cluster correlations. Moreover, all analyses were adjusted for schools as a random effect.

## Results

A total of 5141 out of 7214 school children aged 13 to 14 years completed the questionnaire (response rate: 71.3%). Therefore, our final analysis was done on 5141 children. Demographic information, asthma symptoms and exposure to pets are summarized in Table 1.

**Table 1:** Characteristics of the Study Population in Yazd (n = 5141)

**Characteristics**	**Children, No. (%)**
Gender	
Female	3069 (59.7)
Male	2072 (40.3)
Asthma symptoms& Severe asthma	
Current wheezing	461 (9)
Night dry cough	635 (12.4)
Exercise induced wheezing	816 (15.9)
Severe asthma	102 (2)
Pet keeping	
No animals	3341 (65)
Any animals	1800 (35)
Birds	1390 (27.04)
Birds & cats	110 (2.14)
Birds & dogs	59 (1.15)
Cats	155 (3.01)
Dogs	48 (0.93)
Cats & dogs	13 (0.25)
Cats, dogs & birds	25 (0.49)

Birds were the most commonly kept pets. Moreover, 182 of the subjects kept more than one pet. There was a significant difference between girls and boys in keeping birds as 27.4% of females and 35.8% of males kept birds (*P*: 0.000).

Study-defined current asthma occurred in 108 (2.1%) of our total population study. Of these, 35.18% were pet keepers. The association between current pet keeping (cats, dogs and birds) and current asthma and severe asthma is shown in Table 2. No gender superiority was seen in current asthma and severe asthma in terms of pet keeping. (*P* > 0.05), although boys with severe asthma were more likely to keep pets (*P* = 0.053).

**Table 2:** Association Between Current Pet Keeping (Cats, Dogs and Birds) and Current Asthma and Severe Asthma

**Characteristic**	**Current Asthma**	**Severe Asthma**
Any pet		
Yes	38 (2.1)	49 (2.7)
No	70 (2.1)	53 (1.6)
*P* value	0.97	0.005
Chi-square value	0.001	7.761
Birds		
Yes	31 (2)	45 (2.8)
No	77 (2.2)	57 (1.6)
*P* value	0.63	0.003
Chi-square value	0.230	8.643
Dogs		
Yes	6 (4.1)	4 (2.8)
No	102 (2)	98 (2)
*P* value	0.083	0.497
Chi-square value	3.011	0.460
Cats		
Yes	12 (4)	11 (3.6)
No	96 (2)	91 (1.9)
*P* value	0.02	0.034
Chi-square value	5.414	4.487

Moreover, we evaluated the relations between pet keeping and current asthma symptoms including current wheezing, night dry cough, exercise-induced wheezing, and use of oral medications for relieving asthma symptoms. Further details about the association between current pet keeping and asthma-related symptoms in adolescents living in Yazd and also chi-square values are described in Table 3.

**Table 3:** Asthma Symptoms in the Past Year in Relation to Current Pet Keeping in 13-14 Year-old School Children in Yazd (n = 5141).

**Asthma Symptoms in the Past Year**	**Wheezing ** **No. (%)**	**Night Dry Cough ** **No. (%)**	**Exercise-Induced Wheezing ** **No. (%)**	**Inhaled Medications ** **No. (%)**	**Oral Medications ** **No. (%)**
Any pet					
Yes	191 (10.6)	269 (14.9)	339 (18.8)	66 (3.7)	159 (8.8)
No	270 (8.1)	366 (11)	477 (14.3)	131 (3.9)	231 (6.9)
*P* value	0.002	0.000	0.000	0.65	0.013
Chi-square value	9.17	17.199	18.185	0.205	6.146
Birds					
Yes	164 (10.4)	224 (14.1)	292 (18.4)	52 (3.3)	135 (8.5)
No	297 (8.3)	411 (11.6)	524 (14.7)	145 (4.1)	255 (7.2)
*P* value	0.02	0.009	0.001	0.171	0.091
Chi-square value	5.391	6.774	11.253	1.873	2.865
Dogs					
Yes	20 (13.8)	32 (22.1)	47 (32.4)	16 (11)	21 (14.5)
No	441 (8.8)	603 (12.1)	769 (15.4)	181 (3.6)	369 (7.4)
*P* value	0.000	0.000	0.000	0.000	0.001
Chi-square value	4.257	13.014	30.574	21.005	10.123
Cats					
Yes	44 (14.5)	63 (20.8)	65 (21.5)	19 (6.3)	34 (11.2)
No	417 (8.6)	572 (11.8)	751 (15.5)	178 (3.7)	356 (7.4)
*P* value	0.000	0.000	0.006	0.023	0.014
Chi-square value	12.168	21.188	7.507	5.196	6.069

## Discussion

The prevalence of pet-keeping during the previous year in this study was estimated to be 35% (1800 children), and birds were found to be the most commonly kept pets (88%). Moreover, we detected a significant relationship between any pet-keeping with current asthma symptoms. Another study conducted on primary school children in Isfahan, a city near Yazd, found that the prevalence of keeping birds, cats, and dogs appears to be 26.7%, 5.7%, and 0.5%, respectively.^18^ The roughly similar prevalence of pet keeping in these studies is likely due to the cultural similarity of these two central cities of Iran. Contrary to our findings, bird keeping in that study was only associated with current asthma symptoms.

Further, a case-control study in Tehran revealed that the odds ratio of developing asthma in children who were in contact with a pet was 2.59 times higher than that of the children in the control group.^19^ In addition, as in our study, birds were found to be the most commonly kept pets.

Another study in Zanjan, Iran, which was based on the ISAAC questionnaire, identified a significant correlation between asthma symptoms with exposure to pets during the last 12 months, thus being in line with our results.^20^

Furthermore, in a study by Alshatti and Ziyab, current pet-keeping in Kuwait was reported by 42.8% of the participants. Birds, cats, rabbits, fish, and dogs were among the evaluated pets which were kept by 28.3, 13.2, 7.8, 3.9, and 3.1% of the participants, respectively.^21^ The mentioned study found current cat keeping as significantly associated with current wheezing, which is consistent with our findings. Moreover, they reported that keeping a bird in the house can be associated with current rhinitis and itchy rash. Based on our findings, however, keeping birds and cats was significantly correlated to severe asthma while no association was found between keeping dogs and severe asthma; this could be due to the small number of dog keepers in our region. Unlike our findings, the study in Kuwait failed to find any association between keeping a pet and severe asthma.^21^ Despite the consistent finding that birds were the predominant pets in both studies, the possible difference between the number of birds in the house, the resulting allergic load, method of bird care (number of bird cleaning, and bird house cleaning per week) and housekeeping may be assumed as the reasons for the differences reported in the results. The same is true for cats. However, genetic and climatic differences may also be involved.

Furthermore, we observed a higher prevalence of current asthma among individuals who keep cats as pets. One of the most significant cat allergens is *Felis domesticus*, which can be found in the saliva, anal glands, sebaceous glands, skin, and fur of cats. Due to its small size, it can easily become airborne and persist within indoor environments. These characteristics increase the risk of cat sensitization and allergic reactions. Some researchers have noted that although having cats in the household during childhood appears to be a risk factor for asthma, individuals who have had significant exposure to cats are less likely to develop the disease.^22^ Moreover, cat-keeping in the first year of a child’s life provides protection against asthma and allergic diseases.^23^

In another investigation conducted by Gergen et al, current pet ownership was found to be common in the United States of America, with 51.2% of the participants reported keeping either a dog (37.9%), a cat (27.5%) or both (14.3%) in their houses.^24^ This study concluded that exposure to high levels of dog and cat allergens in people with asthma who are sensitive to these allergens is associated with higher asthma attacks. These findings are consistent with ours, indicating a significant association between severe asthma and pet-keeping.

A global study conducted across 98 countries using ISAAC Phase III findings explored the association between keeping cats and dogs as pets and the occurrence of asthma symptoms and allergic diseases. The research indicated that having current contact with cats, dogs, or both could increase the risk of experiencing asthma symptoms, rhinoconjunctivitis, and eczema among adolescents aged 13 to 14 worldwide.^25^ These findings were consistent with the results of our study which showed that current symptoms of asthma are related to any pet-keeping.

The study by Luo et al in China found that 21.6% of families kept cat, dog, and bird (up to 4%, 14.7%, and 2.5%, respectively). In line with our research, they found that current cat-keeping was significantly associated with diagnosed asthma.^12^ However, unlike our findings, they did not observe any correlation between cat ownership and current wheezing. Despite having a larger number of dog keepers in their study compared to ours, the researchers could not find any association between keeping dogs and current wheezing, which contrasts with our findings.

*Canis familiaris* allergen 1 (Can f1) is known to be one of the main allergens in dogs. This allergen is found in dog hair and dander that can cause hypersensitivity reactions in the airways and skin in sensitized individuals.^26^

The pattern of keeping different types of pets seems to vary according to geographical areas, cultural and even religious differences. Furthermore, the effect of the presence of a pet on the incidence of allergic diseases such as bronchial asthma in different studies varies depending on time and duration of contact, living in urban or rural areas, and heavy or low amount of pet allergens.^20-23,27^

 Our study suffered from several limitations. First, it did not address important questions like “whether or not they had exposure to pets in their first year of life.” Another limitation was reliance on self-completed questionnaires. Furthermore, there were no objective measurements of exposure, although it is known that there is no exact method for measuring pet allergen exposures in observational studies, especially in adolescents who spend much time out of their houses. Moreover, no data on the children’s and their parents’ avoidance behaviors could be collected. On the other hand, the findings were based solely on adolescents’ self-report, and no other methods were used to confirm the presence of allergies, such as the skin prick test or specific immunoglobulin E levels. In addition, confounding factors such as other internal allergens (e.g. cockroaches) and irritants (e.g. smoking) were not considered in this study.

## Conclusion

 In conclusion, our study found a significant association between keeping a cat, bird, or dog and asthma-related symptoms including wheezing, night dry cough, and exercise-induced wheezing. Additionally, cat keeping proved to have a significant relationship with study-defined current and severe asthma. As the most common pet in our area, bird-keeping is a risk factor for severe asthma and the development of current asthma symptoms. Therefore, as an important health tip, this needs to be reminded to families by health care providers. Given the fact that this study evaluated current pet exposure, immunological studies are also required to accurately assess the relationship between pet antigens and asthma-related symptoms.
